# Parental Psychological Control and Depression in University Students: The Chain Mediating Role of Self-Compassion and Emotion Regulation Difficulties

**DOI:** 10.3390/bs15121726

**Published:** 2025-12-12

**Authors:** Keke Chen, Asad Ur Rehman Awan, Tianyong Chen

**Affiliations:** 1 Institute of Psychology, Chinese Academy of Sciences, Beijing 100101, China; kekellychen@163.com (K.C.); asadrehman9400@gmail.com (A.U.R.A.); 2 Department of Psychology, University of Chinese Academy of Sciences, Beijing 100049, China

**Keywords:** parental psychological control, depression, self-compassion, emotion regulation difficulties

## Abstract

Parental psychological control has emerged as a significant risk factor for mental health challenges in young adults. This study aimed to investigate the relationship between parental psychological control and depression, with a specific focus on the chain mediating role of self-compassion and emotion regulation difficulties among Chinese university students. A cross-sectional survey was administered to 363 university students (aged 17–24, 43.3% males, 56.7% females), who completed measures of parental psychological control, emotion regulation difficulties, self-compassion and depression. Findings revealed a significant positive relationship between parental psychological control and depression. Both self-compassion and emotion regulation difficulties independently mediated this association. Furthermore, the chain mediation of self-compassion and emotion regulation difficulties was significant, indicating that parental psychological control was linked with increased depression through decreased self-compassion and increased emotion regulation difficulties. This study sheds light on the significance of minimizing parental psychological control and cultivating a positive family atmosphere to alleviate depression. Parental psychological control has been identified as a potential risk factor for depression, hindering the development of self-compassion, increasing emotional regulation difficulties, with the diminished self-compassion also aggravating emotion regulation difficulties. Despite the cross-sectional design limiting causal inferences, our findings still highlight potential targets for interventions aimed at reducing depression.

## 1. Introduction

Depression has emerged as a pressing global public health concern, drawing increasing attention to the various factors that contribute to its onset and persistence. As the primary environment in which individuals develop, the family plays a pivotal role in shaping mental health outcomes. Parental psychological control refers to a form of control in which parents intrude into their children’s inner world and impede their autonomy development through behaviors such as interference, guilt induction, and love withdrawal (Barber, 1996; Q. Wang et al., 2007). In traditional Chinese culture, authoritative and reciprocal filial piety jointly shape family norms, operating as both independent and interdependent principles (Leung et al., 2010). When authoritative filial piety is prioritized, emphasizing hierarchical parent–child roles and strict obedience, parental psychological control aligns with this cultural expectation (Zhu et al., 2023). Therefore, parental psychological control—a parenting strategy frequently observed in Chinese cultural contexts—has been identified as a positive predictor of depressive symptoms in adolescents (Zhou et al., 2024). A Meta-analysis indicates that psychological control is a globally prevalent negative parenting practice with adverse effects on mental health, observed in both collectivist (e.g., China, South Korea, Ghana) and individualist (e.g., the U.S., Canada, Belgium) countries (Chyung et al., 2022). Although university students may live apart from their families during their studies, advances in communication technologies ensure that familial influence continues to exert an impact. Despite this, much of the existing literature has concentrated on adolescents, with limited research investigating the association between parental psychological control and depression in university students (Y. Li et al., 2024; Rogers et al., 2020; Zhou et al., 2024). Moreover, psychological control has been found to activate maladaptive cognitive schemas and negative belief systems (Swearer & Hymel, 2015), which may not be conducive to the development of self-compassion. Self-compassion—a key aspect of a positive self-concept rooted in secure attachment—has been shown to support emotional regulation and psychological resilience (Gilbert, 2014; Inwood & Ferrari, 2018). However, it remains unclear whether parental psychological control hinders the development of self-compassion, thereby disrupting effective emotional regulation. The present study seeks to examine the relationship between parental psychological control and depression among university students, with a focus on the chain mediating role of self-compassion and emotion regulation difficulties in this association.

As a crucial microsystem for personal development, the family exerts a sustained and enduring impact on individuals, which persists even after students embark on their university life (S. Zhang et al., 2024). As a maladaptive parenting strategy, parental psychological control has been consistently linked to adverse developmental outcomes, including diminished coping abilities, lower psychological well-being, and heightened levels of frustration and anxiety (Deng et al., 2023; Pinquart, 2016; Rogers et al., 2020). Psychological control hinders the formation of healthy attachment relationships, thereby increasing vulnerability to internalizing problems such as depression and anxiety (Guerra et al., 2024; Y. Li et al., 2024). According to the hopelessness theory of depression, psychological control undermines an individual’s perceived control over their internal experiences, contributing to a sense of helplessness, insecurity, and reduced autonomy. These experiences can foster hopelessness, which in turn elevates the risk of depressive symptoms and withdrawal behaviors (Bean & Northrup, 2009). In light of this evidence, the present study hypothesizes that parental psychological control is positively associated with depressive symptoms.

The family environment is closely linked to the formation of self-concept, including the development of self-compassion (Jin et al., 2023). Self-compassion refers to the ability to treat oneself with kindness, understanding, and acceptance during times of emotional difficulty, such as distress, failure, or personal suffering (Neff, 2003b). Neff defines self-compassion as comprising three distinct but interrelated components: (1) self-kindness, as opposed to harsh self-criticism; (2) common humanity, viewing one’s experiences as part of shared humanity rather than as isolating; and (3) mindfulness, which involves maintaining a balanced awareness of painful thoughts and feelings without over-identifying with them (Neff, 2003b). According to the attachment theory, individuals raised in insecure or unsupportive family environments are more likely to internalize external criticism, resulting in the development of negative self-schemas and a diminished sense of self-worth (Bowlby, 1982). Building upon this framework, Gilbert proposed that a secure attachment style facilitates a compassionate self, which effectively balances the functioning of three core emotional regulation systems: the threat and self-protection system, the drive-excitement system, and the soothing-affiliation system (Gilbert, 2010). Parental care provides individuals with a sense of inner security, fostering a feeling of relief and soothing, and enabling them to develop self-compassion (Gilbert, 2020). In contrast, when parental behavior is characterized by neglect, intrusion, or a lack of respect, the environment is perceived as unsafe, leading individuals to be self-critical and consequently over-activate the threat system (Gilbert, 2020; Jin et al., 2023). Therefore, parental psychological control may be a risk factor for the development of self-compassion.

Empirical evidence highlights self-compassion as a protective factor in mental health, associated with reduced levels of psychological distress, anxiety, and depression, as well as enhanced emotional well-being and life satisfaction (Neff, 2023; Zessin et al., 2015). By fostering a non-judgmental and supportive stance toward oneself, self-compassion promotes healthier coping strategies in the face of stress, thereby alleviating depressive symptoms (Shamsababdi & Dehshiri, 2024; Y. Wang et al., 2015). Individuals with greater self-compassion are less likely to be overwhelmed by negative emotional experiences and are more capable of emotional recovery, contributing to greater psychological resilience (Neff, 2003b; Yip & Tong, 2020). Consequently, self-compassion is regarded as a negative correlate of depression, playing a crucial role in adaptive functioning. Within this context, parental psychological control may create a toxic family atmosphere that impairs the formation of self-compassion, ultimately increasing vulnerability to depression. Thus, the current study hypothesizes that self-compassion serves as a mediating mechanism in the relationship between parental psychological control and depression.

Emotion regulation is integral to how individuals process their emotions and foster healthy development, and it is closely related to family environment and self-compassion (A. Finlay-Jones et al., 2017; Martínez et al., 2023). Individuals with effective emotion regulation skills are better positioned to experience greater well-being and social adaptation (Farmer et al., 2023). In contrast, those who face difficulties with emotion regulation are at higher risk of internalizing and externalizing problems (Neumann et al., 2011). Emotion regulation difficulties refer to challenges in emotional awareness, acceptance, and control (Gratz & Roemer, 2004). Research has established a positive correlation between emotion regulation difficulties and depression (Colonnello et al., 2024; Martínez et al., 2023). Individuals experiencing such difficulties often struggle to identify their emotions, lack emotional clarity, and are less likely to accept their feelings (Gratz & Roemer, 2004). Consequently, individuals frequently fail to implement effective emotion regulation strategies when confronted with negative events, which can lead to prolonged immersion in negative emotions and an increased risk of depressive symptoms (Colonnello et al., 2024; Martínez et al., 2023). Furthermore, the use of maladaptive emotion regulation strategies, such as expressive suppression and rumination, extends the duration of negative emotions, thereby exacerbating depression (O’Mahen et al., 2015).

Moreover, parental psychological control impedes the development of individuals’ emotional regulation skills and elevates the likelihood of emotional regulation difficulties (Hope & Chapman, 2019). The development of emotion regulation abilities is deeply rooted in the quality of the early family environment (Martínez et al., 2023). The attachment theory posits that the parent–child relationship forms the foundation for the acquisition of emotion regulation skills (Bowlby, 1982). Children who do not experience a secure attachment are less likely to seek emotional support during times of distress and are more prone to avoidance behaviors, which can hinder the development of effective emotion regulation strategies. Adolescents who maintain positive and supportive relationships with their parents are more likely to engage in adaptive emotion regulation, whereas those from dysfunctional family environments often face greater challenges in this domain (Karreman & Vingerhoets, 2012). Empirical studies further suggest that individuals exposed to adverse familial conditions, such as emotional neglect or abuse, frequently struggle to comprehend and manage complex emotional experiences (Riggs, 2010). Hostile or threatening family environments activate maladaptive physiological responses, such as chronic fight-or-flight reactivity, thereby impairing the development of emotional processing and regulatory systems (Davies et al., 2006; Riggs, 2010). Parental psychological control, characterized by a lack of warmth and emotional support, undermines the formation of secure attachment bonds. This, in turn, contributes to deficits in emotion regulation, which may prolong emotional distress and elevate the risk of depressive symptoms. Based on this evidence, the current study hypothesizes that emotion regulation difficulties mediate the relationship between parental psychological control and depression.

In addition, the link between self-compassion and emotional regulation difficulties suggests that it may play a chain-mediating role between parental psychological control and depression. A growing body of research highlights emotion regulation as a central mechanism through which self-compassion supports mental health. A recent meta-analysis identified emotion regulation as the key pathway by which self-compassion exerts its positive psychological effects (Inwood & Ferrari, 2018). Self-compassion promotes the development of adaptive emotion regulation strategies and is inversely associated with emotion regulation difficulties (Eichholz et al., 2020; Fan & Yang, 2025). A 3-year longitudinal study demonstrated that self-compassion negatively predicts subsequent emotion regulation difficulties (W. Zhang et al., 2025). Further intervention research showed that self-compassion training reduced emotion regulation difficulties and enhanced adaptive emotion regulation skills (A. Finlay-Jones et al., 2017). Self-compassion encourages a balanced and accepting stance toward emotional experiences, fostering self-soothing responses during times of stress or adversity, which in turn enhance emotional regulation capacities (Gilbert, 2014; Neff, 2003a). According to the emotion regulation model of self-compassion, individuals who cultivate a compassionate self-attitude are more likely to engage in non-judgmental awareness and draw upon internal coping resources to manage distress effectively (A. L. Finlay-jones, 2017). On the one hand, self-compassion is positively associated with emotion-focused coping strategies, such as acceptance, which help to reduce negative affect (Allen & Leary, 2010). On the other hand, it promotes a constructive cognitive stance, such as reappraisal, that allows individuals to reinterpret adverse experiences in a less threatening manner (Sirois et al., 2019). Self-compassion reduces excessive identification with negative affect, fostering openness and positive thinking while encouraging acceptance of such emotions within the framework of common humanity, rather than avoidance or rumination (Allen & Leary, 2010; Butz & Stahlberg, 2018; Neff, 2023; W. Zhang et al., 2025). Through these mechanisms, self-compassion may mitigate the emergence of emotion regulation difficulties. Therefore, this study hypothesizes that self-compassion and emotion regulation difficulties jointly function as chain mediators in the relationship between parental psychological control and depression.

In summary, this study explores the relationship between parental psychological control and depression among university students. Parental psychological control hinders the development of self-compassion, which in turn promotes emotion regulation difficulties, increasing depression. Therefore, the following hypotheses and the chain mediation model were established. We hypothesized that parental psychological control is positively associated with depression (Hypothesis 1); self-compassion mediates the relationship between parental psychological control and depression (Hypothesis 2); emotion regulation difficulties mediate the relationship between parental psychological control and depression (Hypothesis 3); self-compassion and emotion regulation difficulties play a chain mediating role in the relationship between parental psychological control and depression (Hypothesis 4).

## 2. Materials and Methods

### 2.1. Participants

This study employed a convenience sampling method, selecting university students from across the country. Using the “two serial mediators” model and the “Set Power, Vary N” setting with 1000 replications and 95% confidence intervals, we employed Monte Carlo power analysis to compute the requisite sample size (Schoemann et al., 2017). The correlation matrix adhered to prior research recommendations. The results showed that at least 153 participants were needed to attain 80% statistical power for the chain mediation analysis. A total of 363 students completed online questionnaires, with 157 males and 206 females, aged between 17 and 24 years (*Mage* = 20.73 ± 1.38 years). Among the participants, 53 were first-year students (14.6%), 94 were second-year students (25.9%), 134 were third-year students (36.9%), and 82 were fourth-year students (22.6%). Additionally, 125 participants were only children, while 238 were non-only children. In terms of residential location, 216 participants lived in urban areas and towns, and 147 lived in rural areas. The study received approval from the ethics committee of first author’s university (H25003). Online informed consent was obtained from all individual participants included in the study, with data collection adhering to Chinese ethical and legal requirements.

### 2.2. Measures

Parental psychological control was assessed using the Psychological Control Subscale from the Parental Control Scale developed by Q. Wang et al. (2007). This subscale comprises 18 items, categorized into three dimensions: guilt induction, withdrawal of love, and power assertion. An example item is, “When I don’t do things the way my parents want, they tell me I should feel ashamed”. A 5-point Likert scale was used, where 1 represented “strongly disagree” and 5 represented “strongly agree”. Higher scores indicate higher levels of psychological control. In this study, the Cronbach’s α coefficient was 0.93.

Self-compassion was assessed using the Self-Compassion Scale developed by Neff (2003a) and revised by Chen et al. (2011). The scale contains 26 items across six dimensions: self-kindness, self-judgment, common humanity, isolation, mindfulness, and over-identification. A 5-point Likert scale was used, with 1 representing “almost never” and 5 representing “almost always”. Higher scores reflect higher levels of self-compassion in the corresponding dimensions. The Cronbach’s α coefficient of this scale was 0.87 in this study.

Emotion regulation difficulties were measured using the Short Version of the Difficulties in Emotion Regulation Scale (Bjureberg et al., 2016). This scale consists of 16 items across five dimensions: non-acceptance of negative emotions, inability to engage in goal-directed behavior, difficulty controlling impulsive behavior, lack of emotion regulation strategies, and lack of emotion understanding. A 5-point Likert scale was employed, where 1 represented “never” and 5 represented “always”. Higher scores indicate higher levels of emotion regulation difficulties. The Cronbach’s α coefficient was 0.93 for this scale.

Depression was measured using the Depression, Anxiety, and Stress Scale (DASS) developed by Lovibond and Lovibond (1995) and revised by Gong et al. (2010). The depression subscale contains 7 items. A 5-point Likert scale was applied, with 1 indicating “never” and 5 indicating “always”. Higher scores reflect higher levels of depression. The Cronbach’s α coefficient for this scale was 0.87 in this study.

### 2.3. Data Analyses

SPSS 22.0 was used to conduct the common method bias tests and correlation analyses. The mean scores of variables were examined for chain-mediated effects using Model 6 in PROCESS macro.

## 3. Results

### 3.1. Common Method Bias Test

Common method bias test was assessed using Harman’s single-factor test. The results revealed that 11 factors had eigenvalues greater than 1, and the first factor explained 27.39% of the variance, which is below the 40% critical threshold. This suggests that common method bias was not a significant issue in this study.

### 3.2. Descriptive Statistics 

Table 1 presents the means, standard deviations, and correlation matrix for the study variables. The results reveal that parental psychological control was negatively correlated with self-compassion (*r* = −0.39, *p* < 0.001) and positively correlated with emotion regulation difficulties (r = 0.48, *p* < 0.001) and depression (*r* = 0.44, *p* < 0.001). When individuals experience heightened psychological control, they have lower levels of self-compassion, along with greater difficulties in emotion regulation and elevated depression. Self-compassion was negatively correlated with both emotion regulation difficulties (*r* = −0.56, *p* < 0.001) and depression (*r* = −0.62, *p* < 0.001). Individuals with high levels of self-compassion show fewer emotion regulation difficulties and lower levels of depression. Emotion regulation difficulties were positively correlated with depression (*r* = 0.67, *p* < 0.001). Individuals with greater emotion regulation difficulties exhibit a higher likelihood of experiencing depression. Given the fact that being an only child and family residence locations are correlated with the main variables, these factors were included as control variables in the analysis.

### 3.3. Mediating Effect Test

**Figure 1:**
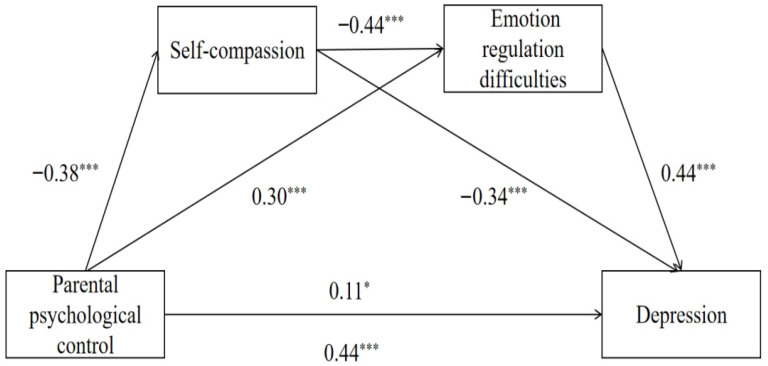
Chain mediating effects of self-compassion and emotion regulation difficulties. * *p* < 0.05, *** *p* < 0.001.

The chain mediation effect was examined using the model 6 of PROCESS macro, with 5000 bootstrap repetitions employed to estimate the 95% confidence intervals. The results, depicted in Figure 1, indicated that parental psychological control positively predicted depression (*β* = 0.44, *p* < 0.001). Even after incorporating the mediating variables, parental psychological control remained a positive predictor of depression (*β* = 0.11, *p* < 0.001). Parental psychological control negatively predicted self-compassion (*β* = −0.38, *p* < 0.001) and positively predicted emotion regulation difficulties (*β* = 0.30, *p* < 0.001). Self-compassion negatively predicted both emotion regulation difficulties (*β* = −0.44, *p* < 0.001) and depression (*β* = −0.34, *p* < 0.001), while emotion regulation difficulties positively predicted depression (*β* = 0.44, *p* < 0.001).

The results of the mediation analysis showed that the indirect effects of self-compassion (*β* = 0.13, 95% CI [0.09, 0.17]) and emotion regulation difficulties (*β* = 0.13, 95% CI [0.09, 0.18]) and the chain indirect effects of self-compassion and emotion regulation difficulties (*β* = 0.07, 95% CI [0.05, 0.10]) were significant. As the 95% confidence intervals for all these indirect effects do not include zero, the indirect effects in this study are significant. The results showed that a higher level of psychological control coincided with lower self-compassion, which in turn was linked to greater difficulties in emotion regulation and higher levels of depression.

## 4. Discussion

This study explored the relationship between parental psychological control and depression, along with the chain mediating roles of self-compassion and emotion regulation difficulties. The results revealed a positive correlation between parental psychological control and depression. The mediating effects of self-compassion, emotion regulation difficulties, and the chain mediation effect were all significant.

### 4.1. Parental Psychological Control and Depression

The study revealed that individuals exposed to parental psychological control are more prone to experiencing depressive symptoms. This finding is consistent with prior research indicating a positive association between parental psychological control and depression (Zhou et al., 2024). Drawing upon the social–ecological diathesis-stress model, parental psychological control can be viewed as a factor that is linked to heightened cognitive vulnerability. This vulnerability is characterized by a child’s tendency to interpret experiences through a pessimistic and self-critical lens, which coincides with greater susceptibility to maladaptive psychological outcomes (Swearer & Hymel, 2015). Through emotional manipulation and intrusion, psychologically controlling parenting is associated with undermining the development of autonomy and fostering maladaptive cognitive patterns, such as rumination—an established risk factor for depression (Hammen, 2018). Moreover, the persistent experience of parental intrusion may be internalized as a sign of diminished personal value and is correlated with a disrupted self-concept and high levels of depression (Y. Li et al., 2024). The link between parental psychological control and depression highlights the necessity of cultivating positive family environments across collectivist and individualist cultures (Chyung et al., 2022). However, it is important to note that the moderate correlation between parental psychological control and adolescent depression underscores the relevance of other factors. For instance, meta-analyses have found negative associations between depression and family-level protective factors, such as resilience and cohesion (Bian et al., 2024; R. Li et al., 2026). Several other risk factors have been linked to depression, such as parental depression, stress, and cognitive dysfunction (Hammen, 2018).

### 4.2. The Mediating Role of Self-Compassion

The present study identified self-compassion as a mediating mechanism in the relationship between parental psychological control and depression. Specifically, higher levels of parental psychological control were associated with reduced self-compassion, which in turn was linked to increased depression. These findings are consistent with the social mentality theory of self-compassion (Gilbert, 2010), which posits that care grounded in warmth, respect, and emotional reassurance fosters a sense of safety and personal value, thereby supporting the development of self-compassion and mitigating negative affect. Conversely, individuals raised in environments marked by parental rejection or family dysfunction are less likely to cultivate self-compassion (Jin et al., 2023). This study contributes to the growing body of literature by identifying parental psychological control as a significant familial risk factor that is linked to impaired development of self-compassion. Psychologically controlling parenting, which is characterized by a diminished sense of child autonomy and worth, is associated with self-doubt and internalized self-criticism, particularly when individuals fail to meet imposed expectations. As an important protective factor, self-compassion has been shown to enhance psychological well-being (Neff, 2023). An Iranian study found a negative correlation between self-compassion and both anxiety and depression (Shamsababdi & Dehshiri, 2024). Meta-analytic evidence supports a robust negative association between self-compassion and depressive symptoms, and intervention studies suggest that cultivating self-compassion can effectively alleviate depression (Ferrari et al., 2019; Hughes et al., 2021). By reducing overidentification with negative emotional experiences and encouraging acceptance rather than avoidance, self-compassion facilitates healthier emotional responses (Yela et al., 2021; Yip & Tong, 2020). Recognizing personal struggles as part of the shared human experience further helps individuals avoid emotional overwhelm, which is correlated with reduced vulnerability to depression (Neff, 2023).

### 4.3. The Mediating Role of Emotion Regulation Difficulties

This study demonstrated that emotion regulation difficulties play a mediating role in the relationship between parental psychological control and depression. Individuals subjected to psychologically controlling parenting practices are more likely to encounter challenges in regulating their emotions, which subsequently increase their vulnerability to depressive symptoms. Consistent with previous findings, parental psychological control has been linked to an increased reliance on maladaptive emotion regulation strategies—such as expressive suppression—and a decreased use of adaptive strategies like cognitive reappraisal (Lai et al., 2014). Such control is associated with disruptions in the development of emotional competence by creating a family environment that is coercive, unpredictable, and emotionally unsupportive (Cui et al., 2014; Hope & Chapman, 2019). Emotional development is largely shaped through early interactions with caregivers. When those interactions are characterized by psychological control, they serve as a negative model for emotion expression and management, which are linked to maladaptive emotional responses and a higher likelihood of persistent emotion regulation difficulties (Morris et al., 2007; Ricker et al., 2024). These regulation difficulties, in turn, are associated with depression. As supported by prior research, emotional regulation difficulties may exacerbate emotional instability and increase the risk of depression (Hollender et al., 2024). Individuals who struggle to identify, understand, and manage their emotional experiences are more prone to experiencing heightened levels of depression (Colonnello et al., 2024; Martínez et al., 2023). Such difficulties are associated with prolonged engagement with negative emotional states, which coincides with negative self-evaluations and more severe depressive feelings (Colonnello et al., 2024). Furthermore, habitual reliance on maladaptive regulation strategies is correlated with more intense emotional distress and disrupted positive affect, factors that are further associated with the development and persistence of depression (Vanderlind et al., 2020).

### 4.4. Chain Mediating Role of Self-Compassion and Emotion Regulation Difficul Ties

Moreover, the chain mediating role of self-compassion and emotion regulation difficulties was supported. Specifically, higher levels of parental psychological control were associated with lower self-compassion, which subsequently linked to greater emotion regulation difficulties, which were in turn associated with heightened depressive symptoms. Similar to a study in the U.S., self-compassion was negatively correlated with emotional regulation difficulties (van Heerden et al., 2025). Longitudinal and intervention studies also provide evidence for the inhibitory role of self-compassion in emotional regulation difficulties (A. Finlay-Jones et al., 2017; W. Zhang et al., 2025). These results align with the emotion regulation model of self-compassion, which proposes that self-compassion is linked to an enhanced capacity for adaptive emotional regulation and a reduction in emotion regulation difficulties (A. L. Finlay-jones, 2017). When individuals are exposed to psychological control, the activation of the threat-protection system is associated with impairments in executive functioning, which may coincide with greater difficulty in regulating emotions effectively. In contrast, self-compassion promotes cognitive reappraisal and emotional recovery by facilitating access to internal regulatory resources, reducing adverse reactions when facing negative affect (Beauchaine, 2015; Svendsen et al., 2016; W. Zhang et al., 2025). Key components of self-compassion—including mindfulness, self-kindness, and recognition of common humanity—are associated with emotional awareness, acceptance, and help-seeking behaviors, all of which are essential for effective emotion regulation (Prentice et al., 2021). Within this framework, individuals with higher self-compassion are better equipped to navigate emotional challenges without becoming overwhelmed, a pattern that is connected to reduced susceptibility to depression. Consequently, the results suggest that parental psychological control is indirectly associated with depression through its linkages with lower self-compassion and greater emotion regulation difficulties, highlighting the importance of fostering self-compassion as a protective factor in emotionally adverse family environments.

### 4.5. Implications

The findings of this study demonstrate that parental psychological control not only is directly associated with depressive symptoms but also is indirectly linked to them through its relationships with lower self-compassion and greater emotion regulation difficulties. These results underscore the observed association of psychologically controlling parenting practices, such as guilt induction and intrusion, and emphasize the need for parents to adopt more supportive and autonomy-promoting approaches. Creating a nurturing, respectful, and emotionally secure family environment is associated with positive psychological development in children and young adults. The identified mediating role of self-compassion suggests that high levels of self-compassion may serve as a protective factor against the negative outcomes associated with parental psychological control. Interventions aimed at cultivating self-compassion have been shown to improve well-being and reduce symptoms of depression (Bluth et al., 2016; Ferrari et al., 2019). Moreover, the established link between self-compassion and improved emotion regulation highlights the potential of self-compassion training for supporting emotional resilience. From a practical perspective, both educational institutions and families play a critical role in promoting emotional competence. Efforts should be directed toward developing programs that foster emotional awareness, acceptance, and the use of adaptive emotion regulation strategies. Such initiatives might help mitigate the psychological correlates of adverse family environments and could be a valuable component in the prevention and alleviation of depressive symptoms among young adults.

### 4.6. Limitations and Future Directions

Despite its valuable contributions, this study has several limitations that should be acknowledged. First, the cross-sectional nature of the study limits the ability to draw definitive conclusions about causality. Although the proposed relationships are grounded in theory and supported by existing literature, the temporal order of the variables cannot be confirmed. Longitudinal or experimental designs would be beneficial in future investigations to more precisely determine causal pathways among parental psychological control, self-compassion, emotion regulation difficulties, and depression. Second, this study employed a convenience sampling approach, which limits the representativeness of the sample across academic disciplines. Notably, we did not collect detailed information regarding participants’ majors or fields of study. Given that students from different academic backgrounds may vary significantly in terms of perceived academic stress, emotional needs, and familial expectations, factors that could potentially moderate or confound the observed relationships, this omission constitutes an important limitation. Future studies should incorporate more diverse and stratified samples to examine how disciplinary contexts shape the psychological experiences under investigation. Third, as college students’ social networks broaden, they build social connections outside their parental sphere and garner social support from varied sources. Future studies should comprehensively investigate the link between social support from diverse sources and their depression.

## 5. Conclusions

The present study demonstrates that when individuals experience more parental psychological control, they are more likely to feel depressed. Parental psychological control hinders the development of self-compassion and intensifies emotion regulation difficulties, while diminished self-compassion further exacerbates emotion regulation difficulties. Collectively, these processes may elevate depression. Although cross-sectional studies are incapable of inferring causal relationships, this research offers potential intervention targets for alleviating depression.

## Figures and Tables

**Table 1:** Descriptive statistics and correlation analysis.

Variables	*M*	*SD*	1	2	3	4	5	6	7
1 Gender	1.57	0.50							
2 Age	20.73	1.38	−0.08						
3 Only Children	1.86	0.48	0.06	−0.02					
4 Residence	1.40	0.49	0.04	−0.05	0.37 ***				
5 Parental psychological control	2.93	0.82	0.01	−0.01	0.05	0.09			
6 Self-compassion	3.18	0.55	−0.05	−0.01	−0.14 **	−0.17 ***	−0.39 ***		
7 Emotion regulation difficulties	2.71	0.84	0.01	−0.02	0.10	0.11 *	0.48 ***	−0.56 ***	
8 Depression	2.15	0.82	−0.001	−0.02	0.05	0.07	0.44 ***	−0.62 ***	0.67 ***

Note: Gender: 1 = male, 2 = female; only children, 1 = yes, 2 = no; residence: 1 = urban areas and towns; 2 = rural areas; * *p* < 0.05, ** *p* < 0.01, *** *p* < 0.001.

## Data Availability

The datasets are available from the corresponding author on reasonable request.
